# Knowledge, attitude, and practice of psoriatic arthritis among patients with psoriasis

**DOI:** 10.3389/fmed.2024.1382806

**Published:** 2024-11-21

**Authors:** Aihua Mei, Mei Luan, Pan Li, Jun Chen, Kuanhou Mou

**Affiliations:** ^1^Department of Dermatology, The First Affiliated Hospital of Xi’an Jiaotong University, Xi’an, China; ^2^Department of Dermatology, Sinopharm Dongfeng General Hospital, Hubei University of Medicine, Shiyan, Hubei, China; ^3^Center for Translational Medicine, The First Affiliated Hospital of Xi’an Jiaotong University, Xi’an, China

**Keywords:** knowledge, attitude, practice, psoriatic arthritis, patient

## Abstract

**Introduction:**

This study aimed to investigate the knowledge, attitude and practice (KAP) of psoriatic arthritis among patients with psoriasis. The KAP questionnaire is a widely used tool in public health research, designed to assess individuals’ understanding (knowledge), beliefs (attitude), and behaviors (practice) related to a specific health condition.

**Methods:**

A cross-sectional study was conducted at Sinopharm Dongfeng General Hospital from September to November 2023. Demographic information and KAP scores were assessed using a structured questionnaire, which evaluated patient knowledge about psoriatic arthritis, their attitude toward managing it, and their practical engagement in preventive or treatment behaviors.

**Results:**

In this study, 392 valid questionnaires were analyzed. Of these, 290 respondents (74.0%) were male, and 296 (75.5%) reported no comorbid conditions. The median scores for knowledge, attitude, and practice were 8 (interquartile range [IQR]: 6–10), 21 (IQR: 19–24), and 14 (IQR: 8–22), respectively. Multivariate logistic regression analysis indicated that practice was independently associated with being female (OR = 0.426, 95% CI: 0.259–0.703, *p* = 0.001), being aged 30–39 years (OR = 2.159, 95% CI: 1.223–3.811, *p* = 0.008) or 40–49 years (OR = 2.002, 95% CI: 1.019–3.936, *p* = 0.044), having a Dermatology Life Quality Index (DLQI) score of 11–30 (OR = 2.569, 95% CI: 1.158–5.700, *p* = 0.020), and not having psoriatic arthritis (OR = 0.300, 95% CI: 0.168–0.537, *p* < 0.001).

**Conclusion:**

Patients with psoriasis had suboptimal knowledge, positive attitude and inactive practice toward psoriatic arthritis. To address this, healthcare providers should prioritize educational interventions, with a specific focus on younger patients, females, and individuals with a higher DLQI score, to enhance awareness and promote proactive management of psoriatic arthritis among this patient population.

## Introduction

Psoriasis, a common dermatologic condition with a global prevalence of approximately 3%, is experiencing an annual increase in incidence (1–4). This chronic disease not only affects the skin but also has significant comorbidities, including psoriatic arthritis (PsA), which is increasingly viewed not merely as a comorbidity, but rather as a distinct manifestation of the same disease with shared pathogenic mechanisms. PsA and psoriasis are interconnected, particularly along the skin-joint axis, reflecting close pathological and immunological links between skin and joint inflammation. Psoriatic arthritis, an immune-mediated inflammatory arthritis, is characterized by peripheral joint inflammation, psoriatic skin lesions, enthesitis, dactylitis, spondylitis, and can lead to progressive disability (5). It impacts a considerable proportion of psoriatic patients, with approximately 5.8 to 30% of those without initial arthritis developing psoriatic arthritis (6, 7). Despite this substantial overlap, there remains a notable gap in understanding how well patients with psoriasis comprehend the risk of developing psoriatic arthritis. This gap extends to their attitudes toward this risk and the practices they adopt in response. Such a knowledge gap highlights the need for further research to better inform patients and guide management strategies.

The Knowledge-Attitude-Practice (KAP) model, a cornerstone in health literacy, plays a crucial role in influencing human health behaviors (8). This model operates on the foundational principle that knowledge positively impacts attitudes, which in turn, shape individual practices (9). To investigate the KAP of psoriatic arthritis among psoriasis patients, we employed a structured KAP questionnaire. It is commonly applied in healthcare research using the KAP questionnaire, a tool designed to comprehensively evaluate the knowledge, attitudes, and practices of a target population. This approach is instrumental in assessing both the demand for and acceptance level of health-related information (10). Despite extensive research on psoriasis and psoriatic arthritis as separate entities, there is a significant gap in the exploration of KAP aspects, particularly concerning psoriatic arthritis among psoriasis patients. This gap presents a critical opportunity to enhance our understanding of how these patients perceive and address the risk of developing psoriatic arthritis. Addressing this gap is vital for devising targeted educational and intervention strategies, aiming to improve both the knowledge and practices of patients. Such efforts are key to achieving better health outcomes and enhancing the quality of life for individuals living with psoriasis and those at risk of developing psoriatic arthritis.

Therefore, this study aimed to investigate the KAP of psoriatic arthritis among patients with psoriasis. Such understanding is crucial for healthcare professionals in refining disease education and facilitating more effective self-management among patients. Addressing the knowledge gaps identified in this study sets the stage for tailored educational interventions, which could substantially diminish the instances of misdiagnosis and delays in treatment, thereby enhancing patient outcomes in the management of psoriatic conditions.

## Methods

### Study design and participants

This cross-sectional study survey was conducted at Sinopharm Dongfeng General Hospital from September to November 2023. The study participants were patients with psoriasis. The study was ethically approved by the Ethics Committee of Sinopharm Dongfeng General Hospital (LC-2023-005) and informed consent was obtained from the study participants.

Inclusion criteria: (1) Diagnosed with psoriasis (Patients must have a clinical diagnosis of psoriasis, confirmed through dermatological examination based on characteristic features such as scaly plaques, typically localized to the scalp, extensor surfaces of the elbows and knees, sacral region, buttocks, and penile area. Involvement of the nails, eyebrows, axillae, umbilicus, and perianal regions may also be observed. The diagnosis should exclude other conditions with similar skin manifestations, such as seborrheic dermatitis, pityriasis rosea, and eczema); (2) 18 years and older; (3) Willing to participate in the survey and capable of understanding the questionnaire; (4) Agree to sign an informed consent form.

Exclusion criteria: (1) Suffering from other types of arthritis, such as rheumatoid arthritis; (2) Severe cognitive impairment or mental illness that prevents understanding or completing the questionnaire; (3) Undergoing medication or other treatments that might affect the survey outcomes; (4) Below 18 years of age or other specified minimum age limits; (5) Pregnant or breastfeeding women, if the study deems this could affect the results.

### Questionnaire introduction

The questionnaire was developed and refined incorporating feedback from 2 experts (2 experts in psoriasis fields). A preliminary version was distributed (50 copies) and demonstrated satisfactory reliability (Cronbach’s alpha = 0.901) and Kaiser-Meyer-Olkin (KMO) measure of sampling adequacy (0.882).

The finalized version of the questionnaire, written in Chinese, gathers data across four dimensions. These include: Basic Information (10 questions), Knowledge (12 items), Attitude (6 items), and Practice (8 items). In the statistical analysis, scoring varies according to the nature of the items. For instance, in the Knowledge dimension, correct responses are awarded one point, while incorrect or unclear responses receive zero points. In the Attitudes and Practices dimensions, scores are assigned on a descending scale from high to zero, reflecting the shift from positive to negative responses. The overall score range is then computed, from the lowest to the highest possible scores. Items not amenable to scoring are treated as distinct categorical variables. Scores exceeding 70% of the maximum possible in each category are indicative of adequate knowledge, a positive attitude, and proactive practices (11).

### Distribution details

This survey is conducted using a combination of paper questionnaires and the Questionnaire Star online survey.

### Statistical analysis

Firstly, the normal distribution test was carried out on the scores of each dimension, and the results showed that the scores of each dimension did not conform to the normal distribution. Respondents’ demographics and dimension scores are described using the median percentile. Score comparisons across different demographics employ Wilcoxon-Mann–Whitney tests for two groups, and Kruskal-Wallis tests for multiple groups. Correlation analysis utilizes Spearman coefficients based on data distribution. The study also conducts univariate and multivariate regression analyses to explore the relationship between demographic data and dimension scores, categorizing results based on average or median values corresponding to the data’s distribution.

In this study, 466 questionnaires were initially collected. However, after a thorough screening process, several were excluded for various reasons: 14 were discarded due to either overly rapid (less than 72 s) or prolonged (more than 1800 s) completion times, 1 was removed due to the participant’s refusal to participate in the study, 2 were eliminated because of completely identical scale answers, and 57 were disregarded due to errors in the questionnaire. Consequently, this left a total of 392 questionnaires that were deemed valid for analysis.

## Results

### Demographic characteristics

Among the psoriasis patients who participated in this study, 290 (74.0%) were male, 187 (47.7%) were aged 30–39 years, 300 (76.5%) had partial reimbursement for medical expenses, 146 (37.2%) had suffered from psoriasis for more than or equal to 15 years, 147 (37.5%) had DLQI scores ranging from 11–30, 271 (69.1%) had PASI score less than 10, and 296 (75.5%) had no comorbidities. The median (25th percentile, 75th percentile) score of knowledge, attitude, and practice were 8 (6, 10), 21 (19, 24), and 14 (8, 22), separately. At the same time, 52.8, 54.6, and 51.5%, respectively, had knowledge, attitude, and practice scores less than or equal to the median (Table 1). Those participants who were younger than 30 years of age (*p* = 0.021), graduated from college/bachelor’s degree and above (*p* < 0.001), had psoriasis for 5–10 years (*p* = 0.014), had psoriatic arthritis (*p* < 0.001), and had greater than or equal to 5 years of duration of psoriatic arthritis (*p* = 0.001) were more likely to have a higher knowledge score. Patients younger than 40 years of age were more likely to have better attitudes relative to 40–49 years of age (*p* < 0.001), while those who graduated from college/university and above (*p* = 0.004) were also likely to have higher attitude scores. When it came to practice, males (*p* = 0.001), those who had psoriatic arthritis (*p* < 0.001), and those who had had psoriatic arthritis for greater than or equal to 5 years (*p* < 0.001) were more likely to have better performance (Table 2).

**Table 1:** Score distribution.

	Median	25% quintile	75% quintile	Minimum value	Maximum value	≤ Median *N* (%)	>Median *N* (%)
Knowledge dimension	8	6	10	0	14	207 (52.8)	185 (47.2)
Attitude dimension	21	19	24	10	30	214 (54.6)	178 (45.4)
Practice dimension	14	8	22	8	40	202 (51.5)	190 (48.5)

**Table 2:** Demographic information and KAP scores.

	*N* (%)	Knowledge (K)		Attitude (A)		Practice (P)	
		Median (25% quartile, 75% quartile)	*p*	Median (25% quartile, 75% quartile)	*p*	Median (25% quartile, 75% quartile)	*p*
Total	392	8 (6, 10)		21 (19, 24)		14 (8, 22)	
Gender			0.109		0.220		0.001
Male	290 (74.0)	8 (6, 10)		21 (19, 24)		15 (9, 23)	
Female	102 (26.0)	8.5 (6, 11)		22 (19, 25)		11 (8, 18)	
Age			0.021		<0.001		0.202
<30 years	80 (20.4)	9 (7, 10)		22 (19, 24)		12 (8, 19)	
30–39 years	187 (47.7)	8 (6, 10)		22 (19, 24)		16 (8, 23)	
40–49 years	76 (19.4)	8 (5.5, 10)		20.5 (19, 23)		15 (8, 22.5)	
≥50 years	49 (12.5)	7 (4, 9)		19 (18, 21)		15 (11, 21)	
Education			<0.001		0.004		0.472
Middle school and below	68 (17.3)	6 (3, 9)		20 (18, 22.5)		14 (8, 19.5)	
High School/Vocational High School	110 (28.1)	8 (6, 10)		21 (18, 23)		16 (9, 22)	
Junior college/Undergraduate and above	214 (54.6)	9 (7, 11)		22 (19, 25)		13 (8, 24)	
Payment methods for medical expenses			0.198		0.857		0.715
Partial Reimbursement	300 (76.5)	8 (6, 10)		21 (19, 24)		14 (8, 23)	
Out-of-pocket	92 (23.5)	8 (5, 10)		21 (19, 24)		14.5 (8, 22)	
Duration of psoriasis			0.014		0.548		0.841
≤5 years	44 (11.2)	7 (4, 9)		21 (18, 24)		15.5 (9, 22.5)	
5–10 years	89 (22.7)	9 (7, 10)		22 (20, 24)		13 (8, 23)	
10–15 years	113 (28.8)	9 (6, 10)		21 (19, 24)		15 (8, 23)	
≥15 years	146 (37.2)	8 (5, 10)		21 (18, 24)		15 (9, 22)	
DLQI score			0.661		0.248		0.181
0–1	36 (9.2)	8 (6, 10)		21 (18.5, 23.5)		11.5 (8, 17)	
2–5	70 (17.9)	9 (5, 10)		21 (19, 24)		12.5 (8, 22)	
6–10	139 (35.5)	8 (6, 10)		21 (18, 23)		14 (8, 23)	
11–30	147 (37.5)	8 (6, 11)		21 (19, 25)		16 (10, 23)	
PASI score			0.139		0.185		0.056
<10	271 (69.1)	8 (5, 10)		21 (19, 24)		13 (8, 22)	
10–20	77 (19.6)	8 (6, 11)		22 (20, 25)		17 (11, 23)	
>20	44 (11.2)	9 (7, 11)		20.5 (17.5, 24)		14 (9, 20.5)	
Psoriatic arthritis			<0.001		0.369		<0.001
Yes	72 (18.4)	10 (8, 11)		20.5 (18.5, 23.5)		18 (13.5, 24)	
No	320 (81.6)	8 (6, 10)		21 (19, 24)		12 (8, 21.5)	
Duration of psoriatic arthritis			0.001		0.121		<0.001
None	320 (81.6)	8 (6, 10)		21 (19, 24)		12 (8, 21.5)	
≤5 years	41 (10.5)	9 (8, 11)		20 (17, 22)		17 (13, 23)	
≥5 years	31 (7.9)	10 (8, 11)		21 (20, 24)		22 (17, 24)	

History of combinations or comorbidities	*N* (%)
a. None	296 (75.5)
b. Cerebrovascular disease (e.g., hypertension)	49 (12.5)
c. Heart disease (e.g., coronary heart disease)	17 (4.3)
d. Diabetes mellitus	20 (5.1)
e. Renal disease	13 (3.3)
f. Malignant tumor	3 (0.8)
g. Other	25 (6.4)

### Knowledge, attitude, and practice

The distribution of knowledge dimension revealed that the two knowledge items with the highest correctness rates were “*Early diagnosis and appropriate treatment of psoriatic arthritis can minimize the occurrence of joint deformities, generally resulting in a good prognosis.*” (K10) with 77.6% and “*Psoriatic arthritis may present symptoms similar to rheumatoid arthritis, other inflammatory arthritis, and gout, requiring careful differentiation for accurate diagnosis.*” (K9) with 73.5%. While the two items with the lowest correctness rates were “*Nonsteroidal anti-inflammatory drugs (NSAIDs) are the most commonly used adjunctive treatment for psoriatic arthritis and can help prevent disease progression.*” (K12) with 7.7% and “*There is no gender difference in the spinal involvement of psoriatic arthritis patients, with an equal male-to-female ratio.*” (K8) with 14.0% (Table 3).

**Table 3:** Distribution of knowledge dimension.

	Correct option
Psoriasis is a chronic inflammatory disease, with approximately 30% of patients with psoriasis developing psoriatic arthritis (PsA).	259 (66.1)
Psoriatic arthritis is highly heterogeneous and is a member of the spondyloarthropathy family.	204 (52.0)
Psoriatic arthritis can occur before the onset of psoriasis skin lesions.	203 (51.8)
Psoriatic arthritis typically occurs only in elderly patients with psoriasis.	284 (72.4)
In most cases, the occurrence of psoriatic arthritis is related to the course of psoriasis.	282 (71.9)
Psoriatic arthritis patients are prone to comorbidities such as metabolic syndrome, cardiovascular diseases, malignancies, and depression.	242 (61.7)
There is no gender difference in the spinal involvement of psoriatic arthritis patients, with an equal male-to-female ratio.	55 (14.0)
Psoriatic arthritis may present symptoms similar to rheumatoid arthritis, other inflammatory osteoarthritis, and gout, requiring careful differentiation for accurate diagnosis.	288 (73.5)
Early diagnosis and appropriate treatment of psoriatic arthritis can minimize the occurrence of joint deformities, generally resulting in a good prognosis.	304 (77.6)
Research indicates that dermatologists may overlook and misdiagnose psoriatic arthritis during diagnosis and treatment, emphasizing the importance of rheumatology consultations to avoid delays in diagnosis.	249 (63.5)
Nonsteroidal anti-inflammatory drugs (NSAIDs) are the most commonly used adjunctive treatment for psoriatic arthritis and can help prevent disease progression.	30 (7.7)

Patients with psoriasis should be alerted to psoriatic arthritis when they present with _______ manifestations and go to the hospital for examination.	*N* (%)
a. Nail damage, punctate pitting, onycholysis, and brown discoloration of the nail bed	218 (55.6)
b. Joint redness and swelling with pain in fingers or toes	239 (61.0)
c. Swelling of soft tissues around the joints of fingers or toes, leading to a sausage-like deformity	231 (58.9)
d. Heel pain and swelling due to inflammation of the Achilles tendon attachment point	181 (46.2)
e. Pain in the lumbar spine or sacroiliac joints	176 (44.9)
f. None of the above	13 (3.3)
g. Not sure	72 (18.4)

**Figure 1:**
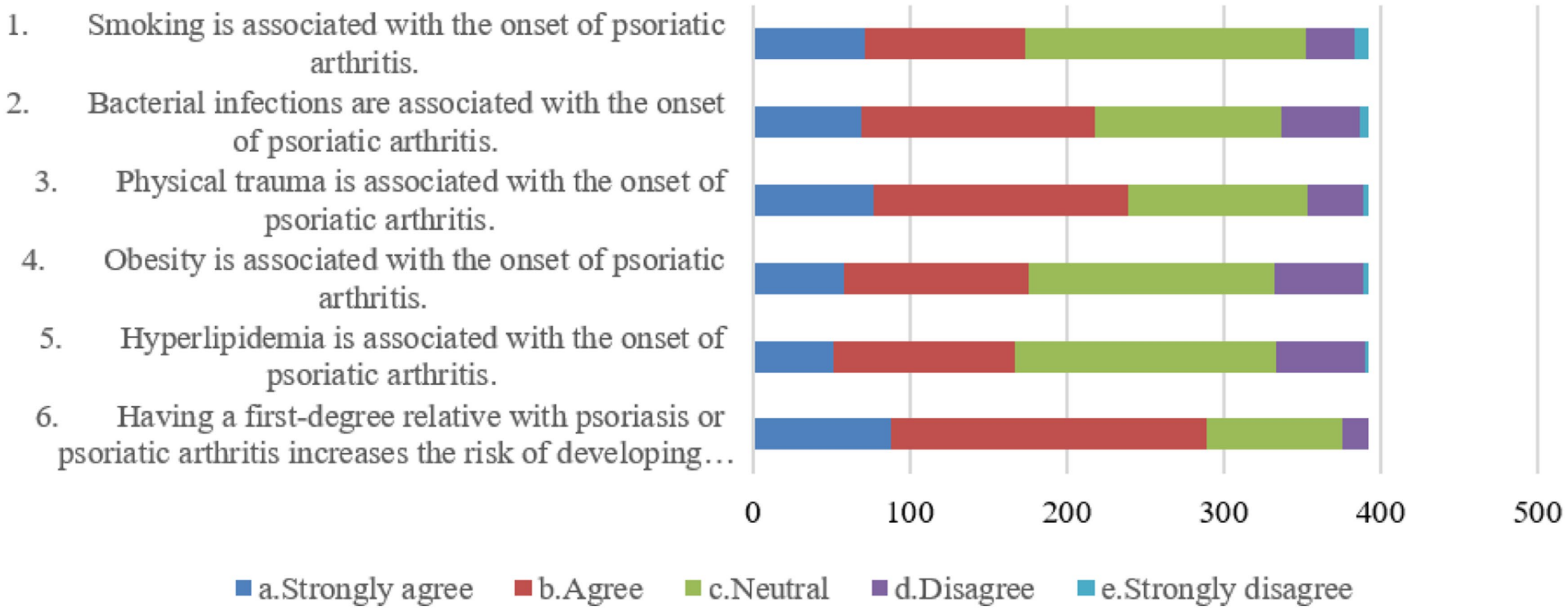
Distribution of attitude dimension.

Patients’ attitudes showed that 38.0 and 41.3% agreed that bacterial infections (A2) and physical trauma (A3) were associated with the incidence of psoriatic arthritis, respectively. Also, 51.3% agreed that patients’ first-degree relatives who have psoriasis or psoriatic arthritis increase the incidence of psoriatic arthritis in patients (A6). Additionally, 45.4, 39.8, and 42.6% were neutral when it comes to the association of smoking (A1), obesity (A4), and hyperlipidaemia (A5) with the incidence of psoriatic arthritis, respectively (Figure 1).

**Figure 2:**
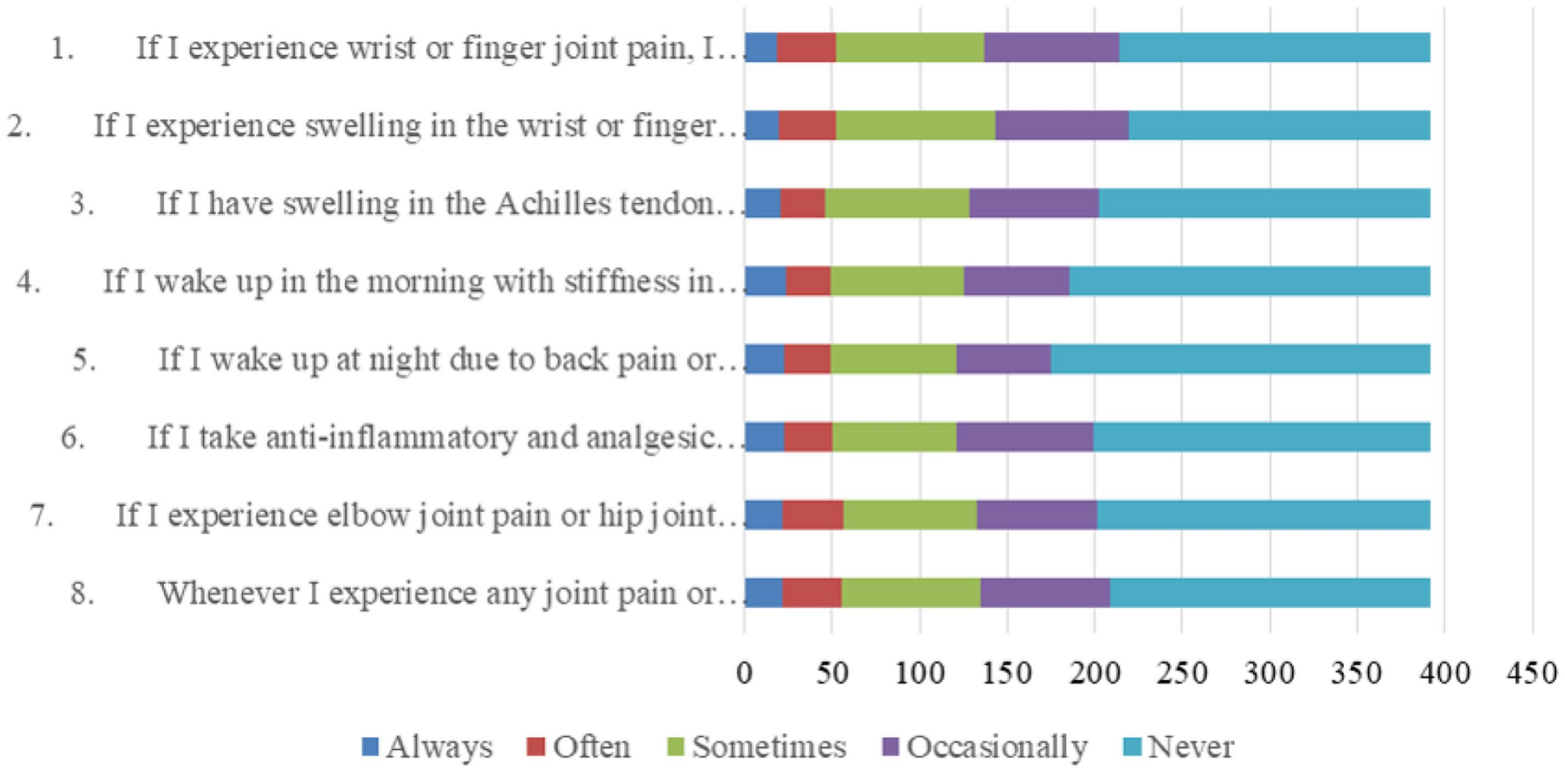
Distribution of practice dimension.

With regard to practices related to examinations or consultations, patients were very negative, with more than 44.1% never performing all these practice items, more than 13.8% performing them occasionally, more than 18.1% performing them sometimes, and no more than 15% performing them always or often combined (Figure 2).

### Correlation analysis and multivariate logistic regression

Correlation analysis showed that there were significant positive correlations between knowledge and attitude (*r* = 0.324, *p* < 0.001) as well as practice (*r* = 0.198, *p* < 0.001). Meanwhile, there was a correlation between attitude and practice (*r* = 0.126, *p* < 0.001) (Table 4).

**Table 4:** Correlation analysis.

	Knowledge	Attitude	Practice
Knowledge	1.000	0.324 (*p* < 0.001)	0.198 (*p* < 0.001)
Attitude	0.324 (*p* < 0.001)	1.000	0.126 (*p* = 0.013)
Practice	0.198 (*p* < 0.001)	0.126 (*p* = 0.013)	1.000

Multivariate logistic regression shown that greater than or equal to 50 years of age (OR = 0.258, 95% CI: [0.114, 0.581], *p* = 0.001), graduated from high School/vocational high school (OR = 2.936, 95% CI: [1.478, 5.832], *p* = 0.002), graduated from junior college/undergraduate and above (OR = 3.143, 95% CI: [1.679, 5.883], *p* < 0.001), and not suffering from psoriatic arthritis (OR = 0.262, 95% CI: [0.145, 0.473], *p* < 0.001) were independently associated with knowledge (Table 5). Meanwhile, 40–49 years of age (OR = 0.503, 95% CI: [0.263, 0.963], *p* = 0.038), greater than or equal to 50 years of age (OR = 0.156, 95% CI: [0.062, 0.392], *p* < 0.001), and graduated from junior college/undergraduate and above (OR = 2.008, 95% CI: [1.112, 3.627], *p* = 0.021) were independently associated with attitude (Table 6). Furthermore, being female (OR = 0.426, 95% CI: [0.259, 0.703], *p* = 0.001), 30–39 years of age (OR = 2.159, 95% CI: [1.223, 3.811], *p* = 0.008), 40–49 years of age (OR = 2.002, 95% CI: [1.019, 3.936], *p* = 0.044), DLQI scored of 11–30 (OR = 2.569, 95% CI: [1.158, 5.700], *p* = 0.020), and not suffering from psoriatic arthritis (OR = 0.300, 95% CI: [0.168, 0.537], *p* < 0.001) were independently associated with practice (Table 7).

**Table 5:** Univariate and multivariate regression analyses for knowledge dimension.

Cut-off value: >8/≤8	No.	Univariate		Multivariate (*p* < 0.1)		Multivariate (*p* < 0.25)	
		OR (95%CI)	*p*	OR (95%CI)	*p*	OR (95%CI)	*p*
Gender							
Male	134/290	ref.					
Female	51/102	1.164 (0.741, 1.829)	0.509				
Age							
<30 years	45/80	ref.		ref.		ref.	
30–39 years	91/187	0.737 (0.435, 1.248)	0.257	0.773 (0.447, 1.339)	0.359	0.773 (0.447, 1.339)	0.359
40–49 years	35/76	0.664 (0.353, 1.248)	0.204	0.645 (0.332, 1.253)	0.196	0.645 (0.332, 1.253)	0.196
≥50 years	14/49	0.311 (0.145, 0.666)	0.003	0.258 (0.114, 0.581)	0.001	0.258 (0.114, 0.581)	0.001
Education							
Middle school and below	19/68	ref.		ref.		ref.	
High school/Vocational High school	53/110	2.398 (1.254, 4.586)	0.008	2.936 (1.478, 5.832)	0.002	2.936 (1.478, 5.832)	0.002
Junior college/Undergraduate and above	113/214	2.885 (1.593, 5.225)	<0.001	3.143 (1.679, 5.883)	<0.001	3.143 (1.679, 5.883)	<0.001
Payment methods for medical expenses							
Partial reimbursement	143/300	ref.					
Out-of-pocket	42/92	0.922 (0.577, 1.474)	0.735				
Duration of psoriasis							
≤5 years	17/44	ref.					
5–10 years	46/89	1.699 (0.814, 3.545)	0.158				
10–15 years	62/113	1.931 (0.948, 3.931)	0.07				
≥15 years	60/146	1.108 (0.555, 2.211)	0.771				
DLQI score							
0–1	16/36	ref.					
2–5	38/70	1.484 (0.661, 3.331)	0.338				
6–10	62/139	1.006 (0.481, 2.104)	0.986				
11–30	69/147	1.106 (0.531, 2.301)	0.788				
PASI score							
<10	124/271	ref.					
10–20	38/77	1.155 (0.696, 1.917)	0.577				
>20	23/44	1.298 (0.686, 2.457)	0.422				
Psoriatic arthritis							
Yes	49/72	ref.		ref.		ref.	
No	136/320	0.347 (0.202, 0.597)	<0.001	0.262 (0.145, 0.473)	<0.001	0.262 (0.145, 0.473)	<0.001
Duration of psoriatic arthritis							
None	136/320	ref.					
≤5 years	26/41	2.345 (1.196, 4.597)	0.013				
≥5 years	23/31	3.890 (1.689, 8.960)	0.001				

**Table 6:** Univariate and multivariate regression analyses for attitude dimension.

Cut-off value: >21/≤21	No.	Univariate		Multivariate (*p* < 0.1)		Multivariate (*p* < 0.25)	
		OR (95%CI)	*p*	OR (95%CI)	*p*	OR (95%CI)	*p*
Gender							
Male	126/290	ref.					
Female	52/102	1.354 (0.861, 2.128)	0.19				
Age							
<30 years	43/80	ref.		ref.		ref.	
30–39 years	101/187	1.011 (0.598, 1.709)	0.969	1.086 (0.638, 1.850)	0.76	1.086 (0.638, 1.850)	0.76
40–49 years	27/76	0.474 (0.249, 0.902)	0.023	0.503 (0.263, 0.963)	0.038	0.503 (0.263, 0.963)	0.038
≥50 years	7/49	0.143 (0.058, 0.357)	<0.001	0.156 (0.062, 0.392)	<0.001	0.156 (0.062, 0.392)	<0.001
Education							
Middle school and below	23/68	ref.		ref.		ref.	
High school/Vocational High school	45/110	1.355 (0.721, 2.543)	0.345	1.611 (0.833, 3.116)	0.157	1.611 (0.833, 3.116)	0.157
Junior college/Undergraduate and above	110/214	2.069 (1.171, 3.657)	0.012	2.008 (1.112, 3.627)	0.021	2.008 (1.112, 3.627)	0.021
Payment methods for medical expenses							
Partial Reimbursement	136/300	ref.					
Out-of-pocket	42/92	1.013 (0.634, 1.619)	0.957				
Duration of psoriasis							
≤5 years	21/44	ref.					
5–10 years	47/89	1.226 (0.595, 2.526)	0.581				
10–15 years	49/113	0.839 (0.417, 1.687)	0.621				
≥15 years	61/146	0.786 (0.399, 1.547)	0.486				
DLQI score							
0–1	17/36	ref.					
2–5	31/70	0.888 (0.397, 1.990)	0.774				
6–10	59/139	0.824 (0.395, 1.720)	0.607				
11–30	71/147	1.044 (0.503, 2.166)	0.908				
PASI score							
<10	119/271	ref.					
10–20	40/77	1.381 (0.831, 2.293)	0.213				
>20	19/44	0.971 (0.510, 1.846)	0.928				
Psoriatic arthritis							
Yes	28/72	ref.					
No	150/320	1.387 (0.823, 2.337)	0.22				
Duration of psoriatic arthritis							
None	150/320	ref.					
≤5 years	14/41	0.588 (0.297, 1.162)	0.126				
≥5 years	14/31	0.933 (0.445, 1.957)	0.855				

**Table 7:** Univariate and multivariate regression analyses for practice dimension.

Cut-off value: >14/≤14	No.	Univariate		Multivariate (*p* < 0.1)		Multivariate (*p* < 0.25)	
		OR (95%CI)	*p*	OR (95%CI)	*p*	OR (95%CI)	*p*
Gender							
Male	153/290	ref.		ref.		ref.	
Female	37/102	0.510 (0.320, 0.811)	0.004	0.426 (0.259, 0.703)	0.001	0.426 (0.259, 0.703)	0.001
Age							
<30 years	28/80	ref.		ref.		ref.	
30–39 years	97/187	2.002 (1.165, 3.440)	0.012	2.159 (1.223, 3.811)	0.008	2.159 (1.223, 3.811)	0.008
40–49 years	40/76	2.063 (1.084, 3.926)	0.027	2.002 (1.019, 3.936)	0.044	2.002 (1.019, 3.936)	0.044
≥50 years	25/49	1.935 (0.937, 3.992)	0.074	1.809 (0.843, 3.881)	0.128	1.809 (0.843, 3.881)	0.128
Education							
Middle school and below	32/68	ref.					
High school/Vocational High school	63/110	1.508 (0.821, 2.769)	0.185				
Junior college/Undergraduate and above	95/214	0.898 (0.520, 1.552)	0.700				
Payment methods for medical expenses							
Partial reimbursement	144/300	ref.					
Out-of-pocket	46/92	1.083 (0.679, 1.729)	0.737				
Duration of psoriasis							
≤5 years	23/44	ref.					
5–10 years	36/89	0.620 (0.300, 1.284)	0.198				
10–15 years	57/113	0.929 (0.463, 1.866)	0.837				
≥15 years	74/146	0.938 (0.478, 1.842)	0.853				
DLQI score							
0–1	13/36	ref.		ref.		ref.	
2–5	29/70	1.251 (0.546, 2.869)	0.596	1.233 (0.516, 2.947)	0.637	1.233 (0.516, 2.947)	0.637
6–10	66/139	1.600 (0.750, 3.411)	0.224	1.667 (0.750, 3.703)	0.210	1.667 (0.750, 3.703)	0.210
11–30	82/147	2.232 (1.050, 4.744)	0.037	2.569 (1.158, 5.700)	0.020	2.569 (1.158, 5.700)	0.020
PASI score							
<10	122/271	ref.					
10–20	46/77	1.812 (1.083, 3.032)	0.024				
>20	22/44	1.221 (0.646, 2.311)	0.539				
Psoriatic arthritis							
Yes	51/72	ref.		ref.		ref.	
No	139/320	0.316 (0.182, 0.550)	<0.001	0.300 (0.168, 0.537)	<0.001	0.300 (0.168, 0.537)	<0.001
Duration of psoriatic arthritis							
None	139/320	ref.					
≤5 years	27/41	2.511 (1.269, 4.968)	0.008				
≥5 years	24/31	4.465 (1.870, 10.661)	0.001				

To further exploring the potential factors that may trigger psoriatic arthritis, both knowledge and attitude items were included as dependent variables in a logistic regression analysis, which showed that K1, K3, K4, and K12, as well as P1, P2, P3, and P8 were independently associated with the triggering of psoriatic arthritis (all of OR > 1, *p* < 0.05) (Table 8).

**Table 8:** Logistic statistics supplement.

Dependent variable: presence of psoriatic arthritis (no = 0, yes = 1)	Content	Univariate	
		OR (95%CI)	*p*
K1	Psoriasis is a chronic inflammatory disease, with approximately 30% of patients with psoriasis developing psoriatic arthritis (PsA).	1.840 (1.020, 3.318)	0.043
K3	Psoriatic arthritis can occur before the onset of psoriasis skin lesions.	1.976 (1.161, 3.366)	0.012
K4	Psoriatic arthritis typically occurs only in elderly patients with psoriasis.	3.136 (1.500, 6.556)	0.002
K12	Nonsteroidal anti-inflammatory drugs (NSAIDs) are the most commonly used adjunctive treatment for psoriatic arthritis and can help prevent disease progression.	3.356 (1.536, 7.329)	0.002
P1	If I experience wrist or finger joint pain, I will go to the hospital for examination.	2.254 (1.171, 4.339)	0.015
P2	If I experience swelling in the wrist or finger joints, I will go to the hospital for examination.	2.254 (1.171, 4.339)	0.015
P3	If I have swelling in the Achilles tendon (back of the foot), I will go to the hospital for examination.	2.172 (1.091, 4.325)	0.027
P8	Whenever I experience any joint pain or swelling, I will seek consultation at the rheumatology department (rheumatology and immunology department) in the hospital.	2.059 (1.076, 3.938)	0.029

Description of score assignment for the questions. Knowledge dimension. K1-K5, K7-K12: correct answers will be scored high, incorrect answers will be scored low. Attitude dimension and Practice dimension: A1-A6, P1-P8: 4 or 5, A1-A6, P1-P8: 4 or 5 are high score, 1–3 are low score.

## Discussion

Patients with psoriasis had suboptimal knowledge, positive attitude and inactive practice toward psoriatic arthritis. Healthcare providers should prioritize educational interventions, particularly targeting older patients, females, and those with a higher DLQI score, to improve awareness and proactive management of psoriatic arthritis.

The present study has uncovered noteworthy disparities in the KAP of psoriatic arthritis among patients diagnosed with psoriasis. It is evident that while the overall knowledge level within the cohort was suboptimal, patients generally exhibited a positive attitude toward psoriatic arthritis. However, their practical engagement and behaviors regarding this condition appeared to be largely inactive. Notably, our results indicated a significant gender-related difference, with females exhibiting a significantly more positive attitude compared to males, despite no significant variance in knowledge levels between the two groups. This gender-based divergence in attitude might reflect distinct healthcare-seeking behaviors and a greater willingness among females to actively engage with health-related measures. These findings align with prior research emphasizing the influence of gender on health-related attitudes (12, 13). Women are often found to be more engaged in healthcare decision-making and more likely to adopt preventive measures, which could explain their higher attitude scores toward psoriatic arthritis (14, 15). However, age emerged as a pivotal determinant, with patients aged 30–39 years demonstrating the highest knowledge and attitude scores, while older patients showed lower scores, indicating a need for focused educational interventions among the older age group (16, 17). Previous studies have similarly reported that older adults tend to have less proactive health attitudes, often due to lower health literacy levels and reduced access to healthcare information (18, 19). Additionally, education level played a critical role, as individuals holding junior college/undergraduate and above degrees displayed the highest knowledge and attitude scores, underscoring the essential role of education in improving awareness and fostering a positive attitude toward psoriatic arthritis.

Moreover, our correlation analyses unveiled significant positive associations between knowledge and attitude, knowledge and practice, as well as attitude and practice. These findings suggest that interventions targeting one dimension may have a cascading effect on the others. For instance, improving knowledge may positively influence attitude, which, in turn, can lead to more proactive healthcare practices. Therefore, comprehensive educational interventions addressing all three dimensions of KAP should be considered.

In addition, our multivariate logistic regression analyses provided deeper insights into factors independently associated with KAP. Notably, older age was associated with lower knowledge levels, while higher educational attainment was linked to greater knowledge and a more positive attitude. The presence of psoriatic arthritis had a negative impact on knowledge, likely due to the focus of patients on their existing condition rather than potential comorbidities. These findings underscore the importance of developing tailored interventions that address the specific needs of different demographic groups, with a particular focus on improving knowledge among older individuals and patients with psoriatic arthritis (20, 21). Additionally, strategies to enhance the knowledge and attitude of patients with lower educational levels should be carefully considered (22, 23). Moreover, our correlation analyses unveiled significant positive associations between knowledge and attitude, knowledge and practice, as well as attitude and practice. These interrelationships underscore the interconnected nature of these dimensions and highlight the potential for interventions targeting one aspect to produce beneficial effects on others.

Furthermore, our study conducted a comprehensive analysis to investigate the factors triggering psoriatic arthritis among psoriasis patients, identifying specific knowledge and attitude components, including understanding the link between psoriasis and joint involvement (K1) and recognizing the importance of early diagnosis and management (P1 and P2), that were independently associated with the occurrence of psoriatic arthritis. In light of these findings, it becomes evident that targeted educational interventions focusing on enhancing patients’ understanding of the intricate relationship between psoriasis and joint complications and emphasizing the significance of early diagnosis and proactive management strategies may hold the key to mitigating the risk of psoriatic arthritis development among individuals diagnosed with psoriasis (24, 25). Such initiatives could potentially serve as preventive measures to reduce the burden of psoriatic arthritis within this patient population.

In comparison to studies conducted in other regions, our findings reflect a similar trend in educational needs and challenges faced by patients with psoriatic arthritis. For instance, Adebajo and Akintayo observed that treatment adherence in psoriatic arthritis can be as low as 57.7%, and effective patient education is crucial for improving adherence, particularly by addressing the disease process, treatment, and self-management strategies (26). Similarly, Sumpton et al.’s review emphasized the psychological and social disruptions experienced by patients, as well as unmet expectations regarding treatment benefits, indicating the importance of addressing psychosocial needs through supportive educational interventions (27). In Nordic countries, Danielsen et al. found that patients with both psoriasis and psoriatic arthritis perceived their disease severity to be greater than those with psoriasis alone, suggesting that both clinical and patient-perceived measures are essential in managing these conditions (28). Additionally, Renzi et al. reported that while many patients with psoriasis or psoriatic arthritis preferred to participate in treatment decisions, their knowledge of treatment options was relatively low, highlighting the importance of patient education to facilitate informed decision-making and proactive health behaviors (29).

Our study reveals a wide range of knowledge levels among participants regarding psoriatic arthritis. While most participants correctly acknowledged psoriatic arthritis as a chronic inflammatory disease associated with psoriasis, a significant number remained unaware of the potential onset of psoriatic arthritis before skin lesions appear. This knowledge gap is concerning, given that early diagnosis is crucial for effective management. Moreover, some participants mistakenly believed that psoriatic arthritis only occurs in elderly patients with psoriasis, which contradicts established knowledge that psoriatic arthritis can manifest across age groups. The highest-scoring item underscores the importance of educating patients about the benefits of timely intervention, emphasizing that early diagnosis and appropriate treatment can minimize joint deformities. These findings align with previous studies indicating knowledge deficiencies regarding psoriatic arthritis among psoriasis patients (30, 31). Addressing these gaps is imperative, and tailored educational programs should prioritize aspects such as the early onset of psoriatic arthritis, dispelling age-related misconceptions, and reinforcing the value of timely interventions to prevent joint deformities (32, 33). Additionally, interventions should emphasize the role of dermatologists in recognizing psoriatic arthritis and the significance of timely rheumatology consultations, which may help mitigate diagnostic delays (34, 35).

The examination of participants’ attitudes toward psoriatic arthritis reveals nuanced perspectives. While some recognize lifestyle factors like smoking, bacterial infections, and physical trauma as potential triggers, others hold neutral or opposing attitudes toward these associations. Interestingly, a substantial number expressed disagreement or neutrality regarding obesity and hyperlipidemia as potential risk factors for psoriatic arthritis. These findings suggest a need for further clarification and education regarding the multifactorial nature of psoriatic arthritis etiology. Furthermore, the highest-scoring item, acknowledging the increased risk with a first-degree relative’s history of psoriasis or psoriatic arthritis, underscores the relevance of genetic factors in psoriatic arthritis. To bridge the gap in attitudes, educational initiatives should emphasize the multifaceted nature of psoriatic arthritis etiology, emphasizing the importance of genetic predisposition while not neglecting other risk factors. Dispelling misconceptions surrounding lifestyle factors and their associations with psoriatic arthritis can lead to more informed attitudes among psoriasis patients (36, 37).

The examination of participants’ practices regarding psoriatic arthritis highlights a range of behaviors. Notably, a significant proportion reported a willingness to seek medical examination in response to specific symptoms, such as joint pain, swelling, and stiffness. However, the consistency of these responses varied across scenarios. While participants displayed a proactive approach to wrist or finger joint pain and swelling, responses regarding lumbar or sacroiliac joint pain were less consistent. This suggests that patient practices may be influenced by the perceived severity or familiarity of symptoms. Furthermore, the highest-scoring item, indicating a willingness to seek rheumatology consultation for joint symptoms, reflects the importance of specialized care in psoriatic arthritis management. These findings emphasize the need for targeted interventions to promote consistent and proactive healthcare-seeking behaviors among psoriasis patients, regardless of symptom location. Educational efforts should stress the significance of rheumatology consultations for timely diagnosis and treatment. Additionally, encouraging patients to recognize the potential seriousness of symptoms associated with different joint sites can foster more consistent practices in seeking medical care (38–40).

This study had limitations. Firstly, as a cross-sectional study conducted at a single center, the findings may not be generalizable to a broader population of psoriasis patients. Additionally, the limited sample size, particularly of patients with psoriatic arthritis, could impact the robustness and clinical significance of the results. A larger and more diverse sample might yield more clinically meaningful insights. Secondly, the majority of respondents were men, creating a gender imbalance that may introduce bias and affect our understanding of patients’ perspectives on psoriatic arthritis. Numerous studies suggest that disease experiences and treatment responses vary by gender, which were not fully captured in this study. Lastly, the cross-sectional design only provides a snapshot of KAP at a specific point, limiting the ability to establish causality or observe changes over time. Future research should consider multi-center studies with larger, more diverse samples, exploring KAP differences across subtypes of psoriatic arthritis and demographic groups. Longitudinal studies could also provide valuable insights into the sustained impact of educational programs on KAP improvement and patient outcomes.

In conclusion, patients with psoriasis had suboptimal knowledge, positive attitude and inactive practice toward psoriatic arthritis. Based on these findings, it is recommended that healthcare providers and educators should focus on improving patient education and awareness about psoriatic arthritis among individuals with psoriasis, particularly targeting younger age groups and those with lower education levels, to enhance their understanding and encourage proactive management and early intervention when needed.

## Data Availability

The original contributions presented in the study are included in the article/supplementary material, further inquiries can be directed to the corresponding authors.
